# Support system diversity among family caregivers of stroke survivors: a qualitative study exploring Asian perspectives

**DOI:** 10.1186/s12877-021-02557-4

**Published:** 2021-10-25

**Authors:** Shilpa Tyagi, Nan Luo, Chuen Seng Tan, Kelvin Bryan Tan, Boon Yeow Tan, Edward Menon, N. Venketasubramanian, Wei Chin Loh, Shu Hui Fan, Kenneth Lam Thuan Yang, Audrey Swee Ling Chan, Aysha Farwin, Zunairah Binti Lukman, Gerald Choon-Huat Koh

**Affiliations:** 1 Saw Swee Hock School of Public Health, National University of Singapore, 12 Science Drive 2, #10-01, Singapore, 117549 Singapore; 2 Policy Research & Economics Office, Ministry of Health, Singapore, Singapore; 3 St. Luke’s Hospital, Singapore, Singapore; 4 St. Andrew’s Community Hospital, Singapore, Singapore; 5 Raffles Neuroscience Centre, Raffles Hospital, Singapore, Singapore; 6 Singapore National Stroke Association, Singapore, Singapore

**Keywords:** Caregivers, Stroke, Social support, Family caregiving, Qualitative

## Abstract

**Background:**

Caregiving is a global phenomenon which is bound to increase in tandem with the aging population worldwide. Stroke is a condition common in older people that requires complex caregiving necessitating provision of adequate support to the caregivers. Past literature consists of limited accounts of types and organization of support arrangements needed by different caregivers. We aimed to describe the support system of caregivers of stroke survivors in Singapore, highlighting differences across the different caregiver identities (i.e. spouse, adult-child, etc.).

**Methods:**

We conducted a qualitative descriptive study in the community setting involving 61 purposively sampled and recruited stroke survivors and caregivers. Semi-structured interviews were conducted, and transcripts were analysed using thematic analysis.

**Results:**

Our findings were summarized across the following 4 themes: 1) cultural influence and caregiving; 2) caregiver support system with the following sub-themes: 2.1) dyadic caregiver support type, 2.2) extended caregiver support type, 2.3.) distributed caregiver support type and 2.4) empowering caregiver support type; 3) breaks in care of stroke survivor and 4) complex relationship dynamics. We operationalized the caregiver support system as comprising of type, people and activities that enable the caregiver to participate in caregiving activities sustainably. While spouse caregivers preferred dyadic and extended support systems positioning themselves in a more central caregiving role, adult-child caregivers preferred distributed support system involving family members with paid caregivers playing a more central role.

**Conclusions:**

Our findings highlight caregiver identity as a surrogate for the differences in the caregiver support systems. Practical implications include imparting relationship-building skills to the stroke survivor-caregiver dyads to sustain dyadic support system and educating clinicians to include differences in caregiving arrangements of stroke survivors in practising family-centred care.

**Supplementary Information:**

The online version contains supplementary material available at 10.1186/s12877-021-02557-4.

## Introduction

The proportion of the world’s population over 60 years will double to 22% in 2050, with an absolute number of 2 billion people over 60 years [1]. Singapore is expected to follow a similar trend of increasing aging population [2]. Caregiving is a global phenomenon, which is bound to increase in tandem with the aging population worldwide. The United States alone accounts for about 52 million caregivers annually [3]. Stroke is a condition common in older people that requires complex caregiving, considering about half of the stroke survivors require some form of assistance with activities of daily living [4]. Often, family members or friends take up this role to support their loved ones who have suffered a stroke, as rightfully described, ‘*caring with love against all odds*’ [5]. About 51% of stroke survivors in the community were reported to receive help from a caregiver, which at times can amount to one full-time equivalent of paid help [6].

Over time, caregiving for stroke survivors may take a toll on the caregivers, as is evident from the existing studies on caregiver outcomes of burden [7], anxiety and depression [8]. Therefore, it is crucial to support the caregivers of stroke survivors to ensure the sustainability of this caregiving arrangement. The first step would be to understand the existing caregiver support arrangements or social support to guide further efforts in this direction. Social support as a broad concept has been described in the context of post-stroke caregiving previously [9–13]. Social support is reported to be associated with lower levels of psychological burden and better adjustment among caregivers of stroke survivors [14, 15]. It empowers the caregiver to provide care in the community and facilitates healthcare navigation by caregivers, specifically flexible support from family and friends [16]. Past literature consists of limited accounts of types of support needed by different caregivers; for example, spouse caregivers expressed the need for formal services like home care, while adult-child caregivers wanted emotional support [17]. However, the organization of such support arrangements, who is providing the support, in what capacity and so forth, remain unexplored. As caregiving experience has been reported to vary across different caregiver types, for example, spouse versus adult-child caregivers [17–19], the types of support arrangements forged may also differ across different caregiver types. This necessitates exploring support arrangements across different caregiver types to find specific gaps or unmet needs and tailor interventions accordingly.

While exploring the support arrangements across different caregiver types it is pertinent to consider the study setting as it is well-established that cultural influences may vary across Asian versus Western settings. The filial obligation is reported to be a common cultural influence in caregiving in Asian settings. It is described as conceptually complex than “*whether or not an individual felt a personal sense of responsibility for his/her parents*” [20]. Furthermore, past literature highlights cultural differences in the perception of caregiving burden, adequacy of social support, satisfaction and use of formal services in Asian settings as compared to their Western counterparts [21, 22]. Within Asian settings, differences in caregiving motivation are reported, specifically adult-child caregivers being influenced by filial piety while spouse caregivers being influenced by culture prescribed obligation to provide care. Another point of divergence in the caregiving context comes from individualism being more common in Western cultures as compared to collectivism being more common in Asian cultures, which has a bearing on the type of support systems available for a family caregiver [23]. This supports the setting specific exploration of caregiver support arrangements to incorporate the implicit cultural influences within Asian or Western settings. Within Asian settings, Singapore presents a unique socio-political context to study caregiving with its hybrid approach to welfare provision ingrained in communitarian ideology, which differs from the individualism observed in Western settings. The prominence of family caregiving in Singapore is rooted in the Singaporean principle of families being the “*first line of support*”, with community and government stepping in where necessary [24].

Our study has potential research, practice and education related implications. Firstly, by addressing the highlighted research gap by describing the organization of support systems, types of support arrangements forged and variations across different caregiver identities, we would be adding new knowledge to existing literature. On the practice front, this new knowledge can inform the development of tailored interventions or programs to address the unmet needs of the caregivers, considering the available support, resulting in the efficient and equitable allocation of resources. On the education front, the findings can complement and inform the curriculum on family-centred management of stroke survivors and their caregivers considering the caregiving ecosystem surrounding the dyad.

## Methods

Within this unique backdrop of Singapore, addressing the highlighted gaps, we aimed to describe the support system of caregivers of stroke survivors, highlighting differences across different caregiver identities (i.e., spouse, adult-child, etc.). Using a qualitative descriptive study design, we conducted semi-structured interviews with stroke survivors and family caregivers [25].

### Participants and selection

We recruited stroke survivors who were Singaporeans or permanent residents, at least 21 years and above, stroke diagnosed by a clinician or supported by brain imaging and were able to participate in the interview. For caregivers, we included individuals aged 21 years and above, who were either an immediate family member, extended family member or friend, were recognized as the main person offering care and taking responsibility for the patient, as recognized by the patient and not paid for caregiving. Recruitment was limited to participants of Asian descent (i.e., Chinese, Malay or Indian). Refusal to audio-record the interview, paid or professional caregivers were the criteria for exclusion. Participants were recruited from an outpatient rehabilitation setting, an outpatient clinic, and a support organization for stroke survivors and their caregivers. The outpatient clinic staff screened their existing patients for eligibility to participate. They shared the list of screened, eligible patients with the study researchers who conducted on-site recruitment during clinic hours with the help of the administrative staff. The physiotherapists or the occupational therapists at the outpatient rehabilitation setting introduced the study to their patients and shared the list of eligible and willing participants with the study researchers who scheduled an interview based on the participant’s preference, at a time and place convenient for them (which could be their homes or community setting). Study information was emailed to the members of the support organization by the relevant staff. Interested members contacted the study research team. Subsequently, interviews were scheduled at a place and time convenient for the participants. Since the study aimed to explore the differences in the description of the caregiver support system across different caregiver identities, we purposively sampled caregivers across different caregiver identities (i.e., spouse, adult-child, sibling and others inclusive of distant relatives or friends). Written informed consent was obtained from the participants. The participants were aware of the scope of work of the interviewers. Our study was approved by the National University of Singapore’s Institutional Review Board (NUS-IRB Ref No: S-18-204). Written informed consent was obtained from all participants after explaining the study purpose and procedures. All research related activities for the current manuscript were performed in accordance with the Declaration of Helsinki.

### Data generation

A total of 61 participants (i.e., 35 caregivers and 26 stroke survivors) were interviewed once from October 2018 to February 2019, at which point thematic saturation was reached. The point of thematic saturation was defined as researchers not observing any new information or themes emerging from the data and this was limited to the overall findings. We could not observe saturation within different caregiver identities as this analysis was only possible after analysing all the collected data, at which point data collection had ceased. Participants were either interviewed at their homes, or outpatient clinic setting or in a community setting of their preference without the presence of non-participants. The sample comprised of 20 caregiver and stroke survivor dyads, 15 caregivers and 6 stroke survivors. The decision to include both dyads and individual participants was guided from a comprehensiveness, feasibility and representativeness perspective. This approach allowed us to recruit caregivers who cared for stroke survivors with severe impairments who could not participate in the study. It was important to capture the perspectives of such caregivers as they may experience tremendous stress, have high caregiving demands and high support needs. The stroke survivors and caregivers recruited as dyads were interviewed separately or together, depending on their preference. Since the accounts shared by participants did not vary depending on whether they were interviewed together or separately, such a distinction was not made during the analysis phase. The interview guide for both caregivers and stroke survivors is detailed in Table 1. These interviews were conducted in the participants’ preferred language (i.e., English, Mandarin, Malay, or Tamil) and generally lasted between 28 to 58 min. The principal investigator (ST), a public health-trained physician pursuing her Ph.D. in health services and systems research at the time of the study, had prior training and experience conducting qualitative research. ST, an experienced qualitative researcher, led qualitative methods and software training for the rest who had basic training in qualitative research (SLC) or were new to it (AF and ZL). While SLC and AF were pursuing their M.P.H at the time of the study, ZL had a B.Sc. and worked as a research assistant at the institution where the study was conducted. All interviewers were female. ST and other researchers involved in data collection (SLC, AF, ZBL) had no prior relationship with the participants. The research team comprised of academics, neurologists, physiotherapists, occupational therapists, research associates, and research assistants, with two members having prior experience caregiving for a family member. Field notes were taken during the interviews and memos were written after the completion of the interview. We also collected socio-demographic information on participants. Audio-recorded interviews were transcribed and translated to English (where applicable). Transcripts were not returned to participants since participant identifiers and contactable information were not retained beyond the interview stage. We removed participant identifiers and assigned an identity code for each transcript to maintain participant confidentiality. NVivo 12 software was used for data management and the facilitation of data analysis [26].

**Table 1:** Interview guide for caregivers and stroke survivors

**Caregiver**	
- How has this new caregiving role affected you? - Do you feel stressed or burdened by the increased responsibilities? Please elaborate. - How do you cope with this caregiving role? - Where do you look for support to help with caring for (stroke patient’s name)? ◦ Probe: Family, foreign domestic worker, friends, neighbours, community networks, formal channels, healthcare providers - What kind of support do you get/expect? - What role do your family members have in caregiving for (stroke patient’s name)? - Could you describe how your culture affects caregiving expectations? - How confident are you in providing care for (stroke patient’s name)? Please elaborate. - Would you say something positive came out of this caregiving experience? Could you elaborate?	
**Stroke Survivor**	
- How do you feel about the care you are receiving from your caregiver? Please elaborate. - Could you describe how your culture affects caregiving expectations? - What role do your family members play in caregiving for you (apart from your caregiver)?	

### Data analysis

We followed Braun and Clarke’s guidance on conducting thematic analysis, which comprised of the following six steps: familiarizing ourselves with our data, generating initial codes, searching for themes, reviewing themes, defining and naming themes and producing the report [27]. Both deductive and inductive coding were adopted with ST as the primary coder, with a subset of interviews being coded by SLC. Coders checked coding consistency via discussions among the team. At each analysis stage, the researchers explored differences across different caregiver identities by constant comparison within and across different groups of caregivers (e.g., spouse versus adult-child). The transcripts were read and re-read thoroughly at the data familiarization step to familiarize ourselves with the data. Comparisons were drawn within and across transcripts of spouse and adult-child caregivers to document preliminary observations of similarities and differences via memo writing. After conducting line-by-line coding of the transcripts to generate a preliminary coding tree, potential patterns and themes were explored by constant comparison at the overall sample level, within subgroups of caregiver identities and across different caregiver identities. The team had discussions on the identified patterns and themes to develop a preliminary understanding of caregiver support systems described along with emerging themes derived from the data. These discussions also incorporated findings at the overall sample and caregiver identity subgroup level to draw meaningful insights from the analysed data. Once the themes and sub-themes were finalized, the differences across different caregiver identities were explored further in-depth by drawing on the discussions and documentation of observations from previous analysis stages and running multiple matrix coding queries in NVivo 12. These matrix coding queries allowed us to explore coding intersections across two items (e.g., analysed coded sections within the transcripts arranged under each theme/subtheme and the categorical variable of caregiver identity) [28]. Please refer to Table 2 for more details. Additionally, we further reviewed the content within each cell of the matrix coding structure for each theme/sub-theme to explore differences and similarities in the narratives given by different types of caregivers under each theme/subtheme. We used the parallel criteria by Lincoln and Guba to guide the development of study processes and report on the trustworthiness of our findings [29]. Credibility or internal consistency was ensured by practising debriefing with peer researchers and co-analysis of a proportion of transcripts with a peer researcher. Peer debriefing was done during data collection to document and discuss insights gained by different team members involved in collecting data and group discussions with peer researchers to share preliminary themes, sub-themes and gain consensus on findings. Under transferability, we provided a clear description of the study context and sample population characteristics so that the reader can assess the transferability of our findings in similar settings. To ensure dependability, we kept an audit trail throughout the conduct of the study (e.g., field notes and memo writing during the conduct of interviews, data analysis and data interpretation using a qualitative data management software, to keep a record of data management and analysis steps taken by the researcher). To ensure confirmability of our findings, the researchers practiced reflexivity, documenting their thought process, perspectives on data collected, and the findings. Also, the authors have shared relevant information on their background, which indicates their epistemological assumptions. Findings are reported in accordance with the COREQ guidelines. (Please refer to Additional File 1).

**Table 2:** Illustration of matrix coding query across different caregiver support system types (coded references) and caregiver identities

	Spouse	Adult-child	Sibling	Others
**1. Caregiver support system:**				
**Theme 1.1. Dyadic caregiver support type**	14	1	0	0
**Theme 1.2. Extended caregiver support type**				
**- Family**	18	9	1	0
**- Friends**	8	10	2	0
**- Faith-based support**	2	1	1	0
**Theme 1.3. Distributed caregiver support type**				
**- Family**	11	47	7	1
**- Foreign domestic worker (FDW)**	14	44	0	0
**Theme 1.4. Empowering caregiver support type**				
**- Informal peer supporters**	39	3	0	1
**- Healthcare providers**	36	29	20	1
**Theme 2. Breaks in care of stroke survivor**	0	25	0	0
**Theme 3. Complex relationship dynamics**	0	24	0	0

## Results

**Fig. 1:**
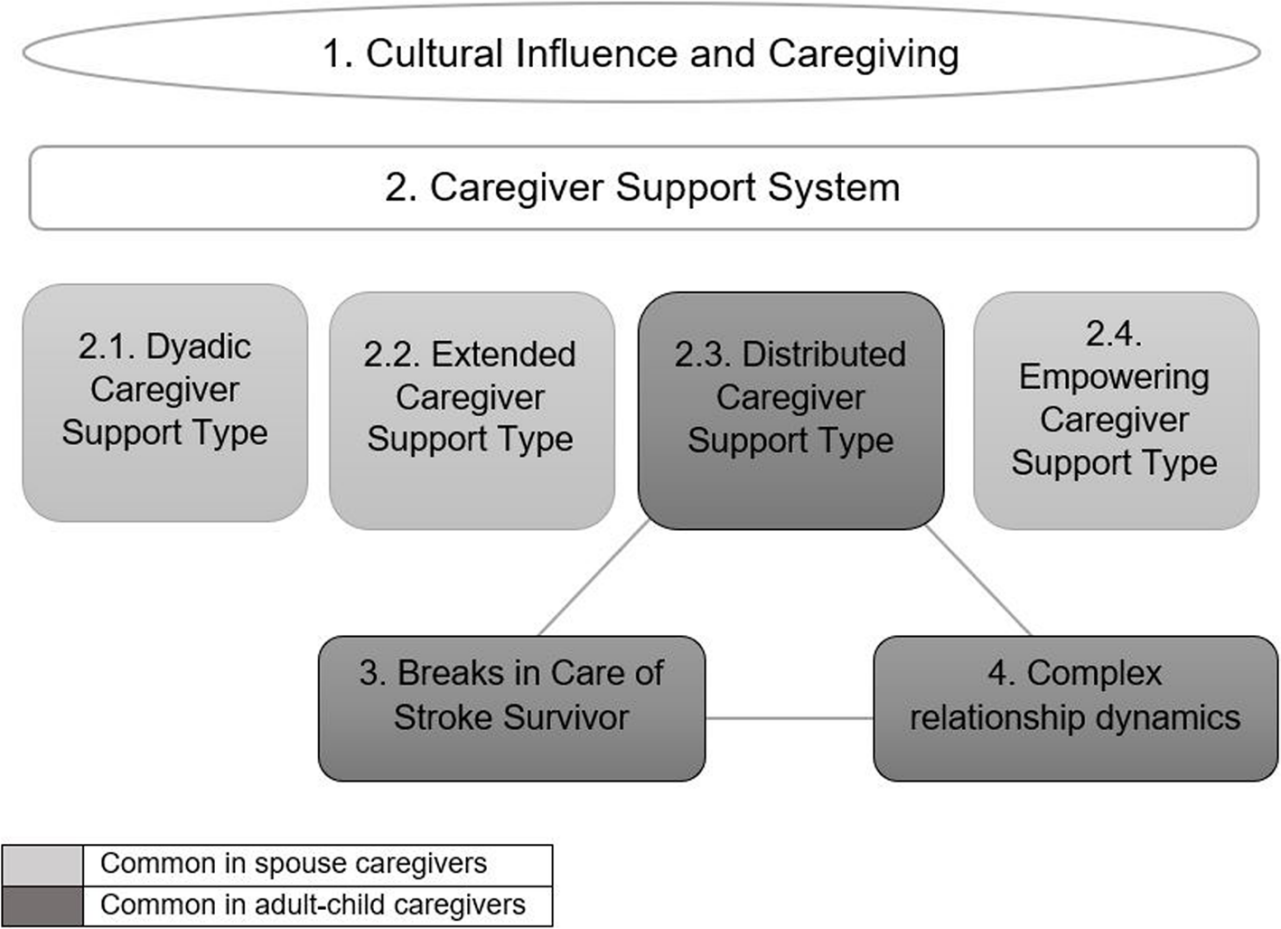
Diagrammatic Summary of Findings

The final sample comprised 35 caregivers and 26 stroke survivors. The non-participation rate was not captured since the preliminary screening and assessment for eligibility criteria was done by staff at the outpatient clinic and outpatient rehabilitation setting who had limited availability of time and resources. Additionally, for the support organization, we were only contacted by participants who were willing to participate. There were 13 spouse caregivers, 18 adult-child caregivers, 2 sibling and 2 other caregivers (Please refer to Table 3). Others comprised of distant family or friends caring for the stroke survivor. Stroke survivors’ age ranged from 45 to 84 years, with 17 males and 8 females among those who provided this information. Most of the spouse caregivers were females (12 versus one), while there was an equal distribution of males and females in adult-child caregivers (10 versus eight). Our sample predominantly comprised of Chinese participants, followed by Malay and Indian. Our findings are summarized across the following 4 themes: 1) cultural influence and caregiving; 2) caregiver support system with the following sub-themes: 2.1) dyadic caregiver support type, 2.2) extended caregiver support type, 2.3.) distributed caregiver support type and 2.4) empowering caregiver support type; 3) breaks in care of stroke survivor and 4) complex relationship dynamics. While the themes of caregiver support system and cultural influence in caregiving were coded both deductively (i.e., derivation of broad categories based on the interview guide) and inductively (i.e., data-driven derivation and refinement of broad categories), the themes of breaks in the care of stroke survivor and complex relationship dynamics were coded inductively and emerged from the data. While the primary focus of the current manuscript was on describing the support system diversity of caregivers of stroke survivors, three additional themes (i.e., cultural influence and caregiving, breaks in care of stroke survivor and complex relationship dynamics) were included to provide a comprehensive context within which such support systems exist. The theme of cultural influence and caregiving was included in the results since it was not only integral to the caregiving experience of caregivers in this Asian setting but also the family members being part of the support system and assisting the caregivers in their caregiving endeavours. The emerging themes of breaks in the care of stroke survivor and complex relationship dynamics were included in the final thematic summary as these are related to the potential sustainability of the distributed caregiver support system, commonly described by the adult-child caregivers. The diagrammatic summary of our findings is presented in Fig. 1 for further clarity.

**Table 3:** Participant characteristics

	Spouse caregiver	Adult-child caregiver	Sibling caregiver	Other caregiver	Stroke survivor
**Total number in each group**	**13**	**18**	**2**	**2**	**26**
**Age (in years)**					
Range	49–80	61–66	51–53	45–79	45–84
**Gender**					
Male	1	10	0	2	17
Female	12	8	2	0	8
**Ethnicity**					
Chinese	6	14	2	1	13
Malay	4	4	0	1	9
Indian	3	0	0	0	3
**Marital status**					
Married living with spouse	13	8	1	1	
Single never married	0	8	1	1	
Divorced	0	1	0	0	
**Education**					
Primary	7	1	0	0	
Secondary	2	4	0	0	
Post-secondary	2	5	1	2	
University	2	7	1	0	
**Employment**					
Working part-time	3	2	0	1	
Working full-time	3	10	1	0	
Unemployed	1	3	1	0	
Homemaker	5	2	0	0	
Retired	1	1	0	1	
**Co-residing with stroke survivor**					
Yes	13	12	2	1	
No	0	5	0	1	
**FDW present**					
Yes	7	12	2	1	
No	6	5	0	1	
**FDW hired for general chores**^**a**^					
Yes	3	7	1	1	
No	4	5	1	0	
**FDW hired for stroke survivor care**^**a**^					
Yes	6	12	2	1	
No	1	0	0	0	
**Number of other family members helping**					
0–1	9	5	1	0	
2–3	4	8	1	0	
4–6	0	4	0	0	

Numbers may not add up to total because of missing values

*Abbreviations*: *FDW* foreign domestic worker

^a^: If answered yes for whether FDW present or not

### Theme 1. Cultural influence in caregiving

Embedded in the caregiving narratives of the caregivers was the role of cultural influence in their caregiving endeavors for their loved ones. Similar to other Asian settings, caregivers in our study shared their motivation for caregiving as familial affection towards their family members or filial obligation towards their parents or elderly family members. While familial affection was commonly observed in the narratives of both spouse and adult-child caregivers, filial obligation was observed in the narratives of few adult-child caregivers. Under familial affection, caregivers shared that their caregiving efforts were directed towards ensuring the well-being of their loved one (i.e., stroke survivor) and preventing another stroke from occurring.

*No, because he is family. If there is anything I would just tell him (..) The more he feels worse, the harder it feels (..) And when he is sick you wish for him to quickly get better.* (C06, 64 years, female, spouse caregiver)

Similarly, adult-child caregivers shared that they cared for their parents and would engage in caregiving to ensure their parents were well, healthy and happy. One of the adult-child caregivers shared that she would let go of opportunities to socialize with her friends to look after her father (i.e., stroke survivor) and viewed this as an opportunity to get closer to her father and strengthen their relationship further. Another shared that it was important to ensure their parents were taken care of even if the caregiver had to make sacrifices as they did not “*want to live with regrets*”.

*Take care of her more than that. If possible, love her don’t make her hate you or make her cry and be hurt. Don’t make her sad. Always make her happy. Because your mother is your heaven (..) Yeah. Because she gave you life and she nurtured you. You know your mother; you cannot see another one in the world.* (C03, 58 years, male, adult-child caregiver)

Stroke survivors were considered as a member of the family unit and family was expected to help with caregiving as is experienced in other Asian cultures. Hence, cultural influence not only impacted the caregiving experience of family caregivers but was also important in family members assisting the caregivers and being part of their support system.

*Yes, family comes first. A family has to take care lah*. (C08, 59 years, female, spouse caregiver)

Filial obligation included accounts of adult-child caregivers describing caregiving as their duty, expectation from others, or something they had to do without any choice. One of the adult-child caregivers shared that by caring for his parent, he was setting an example for his son to do the same if he had a similar misfortune in the future. Few shared that it was essential to provide care to their parents as it is expected from a cultural perspective and is also considered responsible.

*The Chinese thinking is that the daughter, as daughter yeah, you should care for your parents much yeah*. (C25, 50 years, female, adult-child caregiver)

Another adult-child caregiver shared that she had no choice but to care for her mother, as illustrated by the quote below:

*So, I have to do … because my sister is really providing for rest of the family. So, I have got no choice (..) In terms of progress, my mom's progress, we have … it’s really very, very really slow. I mean … I hate to say it but, I mean, to myself I see it's just going to be like that. Of course, there's nothing you can do. I mean she's my mom. We have to keep on providing her support until whatever whenever it is.* (C16, 53 years, female, adult-child caregiver)

### Theme 2. Caregiver support systems

We described the caregiver support system as comprising of type, people and activities that enable the caregiver to participate in caregiving related activities sustainably. This could include paid, unpaid or both types of caregiving arrangements. While unpaid caregiving arrangements comprised of family (including the stroke survivor), friends and community (including faith-based and informal peer support), paid caregiving arrangements comprised of foreign domestic workers (FDWs). FDWs are waged female domestic workers paid a fixed monthly salary to provide services within the household employing them. They assist with household chores, and in some instances, with caregiving related tasks. FDWs are mandated by the Ministry of Manpower in Singapore to reside in their employer’s house under a strict legal permit system [30]. Based on the constituting people, activities, caregiving arrangements and settings, we described four sub-themes depicting the following types of predominant caregiver support systems: dyadic caregiver support type, extended caregiver support type, distributed caregiver support type and empowering caregiver support type (Please refer to Table 4 for more details). Caregivers reported family as the most common source of support and family members were categorised as part of dyadic, extended and distributed caregiving support types. While the spouse caregivers mostly described dyadic and extended caregiver support types, adult-child caregivers mostly described the distributed caregiver support type involving the family. Informal peer support and faith-based support constituted part of the empowering and extended caregiver support types, respectively.

**Table 4:** The caregiver support system of family caregivers of stroke survivors

1. **Types**	**Dyadic caregiver support type**	**Extended caregiver support type**	**Distributed caregiver support type**	**Empowering caregiver support type**
2. **Paid or unpaid**	Unpaid	Unpaid	Paid/ Unpaid	Unpaid
3. **People** constituting support system (along with the family caregiver)	Stroke survivor	Family, friends, community (e.g., faith-based)	Family (mainly siblings), foreign domestic worker or FDW	Peer support group, healthcare providers
4. **Activities** included in the support system	Emotional support and mutual understanding	Complementary support with activities, including but not limited to visiting the stroke survivor and providing company, accompanying the caregiver for shopping, helping with the household chores, or lending a listening ear	All caregiving related tasks	Sharing information, gaining knowledge and skills, the avenue for getting new ideas, support and advice from peer caregivers who were more experienced
5. **Setting:** community or healthcare setting	Community	Community	Community/ Healthcare	Healthcare
6. **Caregiver identity**: most commonly coded descriptions from	Spouse caregivers	Spouse caregivers	Adult-child caregivers	Spouse caregivers

The caregiver support system is operationalized as comprising of types, people and activities that enable the caregiver to participate in caregiving related activities sustainably. This could include paid, unpaid or both types of caregiving arrangements. Four types of caregiver support system are reported: dyadic caregiver support type, extended caregiver support type, distributed caregiver support type, and empowering caregiver support type. The following are the attributes described across each caregiver support type: paid or unpaid caregiving arrangement, constituting people, activities included, setting of operating of the support system and caregiver identity type most commonly coded descriptions came from

### Sub-theme 2.1. Dyadic caregiver support type

This was based on the mutuality of emotional support between the stroke survivor and caregiver dyad. Few participants described the need for the stroke survivor to support their caregiver, often the spouse providing care. We coded this reciprocity of support expressed by caregivers and stroke survivors under the dyadic caregiving support system. Caregivers acknowledged that providing care for a stroke survivor was not an easy task, and the stroke survivor should also realize and support their caregiver in this endeavour. Similarly, the stroke survivors who expressed their views on this acknowledged that caregivers should be given due credit and supported where possible.

*I realize it. Uhm, it is equally- it is equally important to take care, not just the stroke patient, but also the caregiver. (..) The caregiver is the uh, is the root which without it, you (stroke survivor) are down. (..) We realize that we need each other. (..) Yeah, because some patients, they disregard the caregiver, uh, which is wrong because the caregiver is actually aiding you in your recovery. Uh, if you do not, if you disregard them, then you are just um, putting a stop to your uh, movement, your advancement, everything.* (P15, 49 years, male, stroke survivor)

### Sub-theme 2.2. Extended caregiver support type

Extended caregiver support comprised of instances where the caregiver was the main provider of care with complementary support, in the form of cordial visits from family or friends checking on the dyad’s well-being. It was more commonly coded in the interviews of the spouse caregivers where other family members or friends provided complementary support with activities like visiting the stroke survivor and providing company, accompanying the caregiver for shopping, helping with the household chores or lending a listening ear.

*Sometimes yeah. Sometimes I need to talk, uh my problem. All-day I am at my working place. Be at home also sometimes have problems about my maid. Sometimes, problem with my maid also, right? Sometimes, lah. Right. She’s good. I can talk to my daughter lah*. (C08, 59 years, female, spouse caregiver)

Stroke survivors expressed similar views with family members and friends providing extended support in spending time with the stroke survivor at home or accompanying the stroke survivor on outings to help lighten their mood.

*Sunday, we go out. Go to my brother’s place (..) When spend the morning, afternoon, all day in Malaysia out there. I went all day in Malaysia and then only take the car to go, drop passport and then take bus and go makan. Lunch, dinner and then come back very late*. (P18, 68 years, male, stroke survivor)

Some of the caregivers reported friends as constituents of their extended caregiver support system. Few caregivers reported finding support in a faith-based organization which was coded under extended caregiver support system.

### Sub-theme 2.3. Distributed caregiver support type

Distributed caregiving comprised of instances where participants explicitly stated in their descriptions that caregiving was a shared activity among family members: ‘*we take turns*’, ‘*help in planning care*’ or ‘*we pool resources*’. Caregivers either proactively sought to build such a distributed caregiver support system or let them develop organically. Such caregiving arrangement was more commonly coded in interviews by adult-child caregivers where other family members, usually siblings, participated in caregiving with shared responsibilities, resources, and funds.

*So... So, I have three younger brothers, so there are four of us. So that is a good thing. We can split the job up, but I am the eldest, so I will be the one leading. Someone has to take the lead, so I am taking the lead. I won’t say that I am the primary caregiver, but I am the primary person to take over all those things now. (..) Of course, we all have a WhatsApp group chat, and we coordinate from there. Our motto is (when it comes to caregiving) ...We do – how do I say, how would I put it? - we all contribute to the best of our ability, so we don’t compare.* (C21, 50 years, male, adult-child caregiver)

A quote from one of the stroke survivors further illustrates how family members identified their strengths and distributed the caregiver duties among themselves to sustain their caregiving arrangement:

*Correct. That’s why I think we are also fortunate because we (family members) have identified whose strength plays what role (in caregiving).* (P13, 45 years, female, stroke survivor)

The FDWs constituted the paid component of the distributed caregiving support type of the family caregivers considering they are waged domestic workers who are paid a monthly salary for performing household chores and providing specific caregiving assistance to the stroke survivor. The FDWs’ assistance varied with different care contexts; for example, they could help with self-care activities of the stroke survivor, transport, medication management or enable family caregivers to continue working by assisting with competing household chores.

*I have another helper to look after her on a daily basis, you know to cook for her and to shower her to look proper, to give her medication and all that (..) Without the helper then it would be more challenging. I’ll have more worries. Who is going to give her medication? Who is going to give her, to cook her meals or shower her and all that? With the helper it helps a lot.* (C29, 54 years, male, adult-child caregiver)

### Sub-theme 2.4. Empowering caregiver support type

Empowering caregiver support system was comprised of instances where the caregiver gained information, knowledge or skills that supported their caregiving role. Spouse caregivers more commonly reported the support received from informal peer support groups than adult-child caregivers. The outpatient rehabilitation therapy appointments were reported as opportunistic touchpoints to interact with peer caregivers for getting new ideas, support and advice.

*I did identify his like, “Buy this.” Then help out with advice about hand, that time we had the thing for rubber band. (- -) Yeah, things like because she was telling me her husband can go to the toilet on his own, I said, “You must be very careful because from what I see, he’s not that stable yet.” He’s not that stable yet. So, other than that, like the longer I sit with a person. The rubber for hand, I got it from resident whose mother not using. I pass it to her since we are not using. Because her husband’s pay will be cut in half over six months, maybe. So, I understand financially will be a problem. She got two kids. One is working; one is not. I think now she stuck with the bill. So somehow, I understand her situation and at that time when she came, she was very down. It really reminds me of me when I was in that situation.* (C15, 45 years, female, spouse caregiver)

Participants also made friends and shared light moments, which helped them in their post-stroke caregiving journey. Stroke survivors also shared similar views and expressed how interacting with other stroke survivors helped them in their recovery journey.

*Yes, he (fellow stroke survivor) is older. He was telling me, “Be strong, exercise...” He does his TV exercises... So to me that was a role model - that you could get up.* (P17, 57 years, male, stroke survivor)

### Theme 3. Breaks in care of stroke survivor

This was the first emerging theme which we coded at the intersection of paid and unpaid caregiving arrangements of the caregivers. To further elaborate, this theme evolved from the description of the interactions caregivers had with their hired FDWs, with caregivers and their family members considered under unpaid caregiving arrangement and FDWs as their paid counterparts. Interestingly, this theme of breaks in the care of stroke survivor, as well as the subsequent theme of complex relationship dynamics, were exclusively described by the adult-child caregivers. This is understandable as FDWs were described under the distributed caregiver support system, which was more common among adult-child caregivers. This theme included instances resulting in a discontinuity in the caregiving assistance provided by FDWs. Discontinuity was reported at four levels: (1) frequent weekly, (2) annual, (3) end of term and (4) sudden discontinuity. Frequent weekly discontinuity referred to the weekend off all FDWs are entitled to in Singapore. Annual discontinuity in care referred to the period of leave (about 2 weeks) the FDWs are entitled to, during which they often return to their home country, leaving the family caregiver in charge of making alternative care arrangements. End of term discontinuity included instances illustrating the high turnover rate of FDWs as they are hired on a term contract, after which they usually return to their home country, leaving the family caregiver to find a replacement.

*But to be honest, in the last four years, I had four to five helpers not because I changed them (of course there is that one that I sent back) but because their contract was up, so they needed to go back and get the new one. You know, after my mom passed away, one of them wanted to go back, and the other one wanted to renew it, so eventually, they both went back. The one that I have right now I will say is the best of all that I have or had in the past. (..) Yes, yes, so this other, we have to make sure that we call (in advance) or we cope with it.* (C21, 50 years, male, adult-child caregiver)

Few adult-child caregivers described experiences of sudden discontinuity, in which case, the FDW would stop working unexpectedly, leaving the family caregiver to get immediate respite or substitute.

*It’s just crazy. There isn’t a place that I can go to. The three of us we have training next week. I can’t stay home, and my sister can’t do all this (caregiving), so I don’t know how we will manage.* (C28, 43 years, female, adult-child caregiver)

All of these breaks in the continuity of care highlighted the transient nature of the paid caregiving arrangement in the support system of the family caregiver.

### Theme 4. Complex relationship dynamics

Some adult-child caregivers described the complex relationship dynamics they shared with the FDW caring for their stroke survivors. They highlighted feeling helpless, recognizing the tricky situation they were in. Since they could not oversee the care delivered by the FDW, they could not stress the FDW as it may have direct repercussions on their loved one being cared for by the FDW. They shared that they would consciously let go of certain things and not scold the FDW as they were uncertain how she might respond to critique or stress. As one caregiver described, ‘*You have to cover your eyes on her other flaws so long as she can take care’.*

*Then what he’ll (father of caregiver) do, he’ll be like saying that’s wrong … I mean, he is not wrong, and he has the right to do it but … I’m just saying … I’m just trying to see from another perspective is that it might not be a good way to keep doing that, you know helpers right, you don’t know what they are thinking. We depend on them, it’s not as easy as this is not good, and oh we can change. This is not something we want to (do) also because eventually, it may not be good in the long run.* (C16, 53 years, female, adult-child caregiver)

Some of the caregivers managed this situation by trying to build trust with the FDW and motivating her to take better care of the stroke survivor.

*I would say after a few years you just have to trust things. You cannot be restrictive. In terms of the food she prepares for her, it’s also pretty much the same on a daily basis. She can’t cook well. It’s a simple vegetable or steamed fish or soup.* (C29, 54 years, male, adult-child caregiver)

## Discussion

We are the first to the best of our knowledge to describe the support system diversity of caregivers of stroke survivors in an Asian setting. Additionally, we contributed new knowledge to existing literature by highlighting the differences in key themes across the spouse and adult-child caregivers. Specifically, we described the support system of caregivers of stroke survivors comprising of the following four sub-thematic types: dyadic caregiver support, extended caregiver support, distributed caregiver support, and empowering caregiver support. Additionally, we described the cultural influence in caregiving in the Asian setting of Singapore and the following two emerging themes: breaks in care of stroke survivor and complex relationship dynamics.

We found cultural influence not only impacting the caregiving experience of family caregivers but also family members being part of the support system of caregivers. In fact, family members were reported as the most common source of support which is aligned with the ideology of family being “the first line of support” in Singapore [24]. The findings within the theme of cultural influence and caregiving were aligned with the previous literature in familial affection and filial obligation driving the caregiving endeavours of caregivers [23].

While the description of the four predominant caregiver support systems was one of the unique contributions of the current study, additionally, reporting of FDWs being part of the distributed caregiving support system and playing a relatively more important role in post-stroke caregiving in the narratives of adult-child caregivers was another novel finding. In fact, FDWs were mentioned as part of the caregiver support system almost exclusively by the adult-child caregivers, which highlights the more significant role of FDWs in caregiving of stroke survivors involving adult-child caregivers. One of the explanations of the different roles of FDWs across caregiver support systems of spouse versus adult-child caregivers could be related to their different perspectives towards FDW. While assistance from FDW has been reported to be associated with a reduced amount of caregiving and a lower negative reaction to caregiving, it is associated with lower self-esteem in spouse caregivers only, which implies, they may be less willing to involve FDWs in a more central caregiving role as opposed to adult-child caregivers [31].

We reported 2 emerging themes which may potentially impact the sustainability of the distributed caregiving support system, one of which was breaks in the care of the stroke survivor. A systematic review synthesizing qualitative literature from 51 papers involving 328 caregivers and 168 stroke survivors reported findings in concordance with this theme, especially for those reliant on paid helpers. This review highlighted the need for supporting caregivers to tide over emergencies and break in the care of the stroke survivors [32]. However, the involvement of paid helpers or FDWs in the family caregiving arrangements is not just limited to challenges or difficulties; it also presents an opportunity for adult-child caregivers to cope with competing commitments and participate in caregiving duties post-stroke. Availability of paid helpers or FDWs made it possible for adult-child caregivers to have this unique caregiving arrangement, whereby they delegated part or most of the physical aspects of caregiving to the FDW, who usually resided with the stroke survivor under the distributed caregiving support type. This allowed the adult-child caregiver to tend to other roles and responsibilities on the family and work front. However, such an option may not be available in all settings; for instance, a qualitative study in Canada involving adult-child caregivers reported caregivers having limited or no choice to distribute caregiving responsibilities. They often had to compromise on other fronts to fulfil their caregiving role [33]. Another study from the UK reported caregivers feeling trapped in their predicament with limited support from family or friends to assist with the caregiving role [34].

While FDWs were part of paid caregiving arrangements under the caregiver support system, family (including stroke survivors), friends and community were part of the unpaid caregiving arrangements. A qualitative study involving spouse caregivers of stroke survivors shared the importance of support in the form of help from family, friends or others for stroke survivor-caregiver dyads to adjust in the community setting. However, the focus was limited to exploring caregiver needs post-stroke rather than providing a comprehensive description of the support avenues to address such caregiver needs. The importance of studying and reporting of such support systems is further highlighted by existing literature recommending assessment of the support system in the community as part of the caregiver needs assessment post-stroke [13]. While authors have previously reported the importance of having such support in the form of assistance from family members [12], there have been limited efforts towards a comprehensive description of the caregiver support system and its attributes, and our findings are timely in addressing this existing gap in literature. Similar to our finding of unpaid caregiving arrangements comprising of different sources of support, a meta-synthesis involving 12 qualitative studies reported the importance of support for caregivers post-stroke from different sources, comprising of assistance with different caregiving related tasks [35]. Along the same lines, a qualitative study in Sweden exploring the lived experience of being a close relative of someone who had a stroke described the importance of support by different sources like peer supporters, friends and family with whom the caregiver could offload. Additionally, healthcare providers were also described as avenues for addressing caregiver’s needs [9].

The caregiving support systems were not only based on different sources of support from family, friends, community and paid helpers but also from the stroke survivor as described under the dyadic caregiver support type. In concordance with our findings of caregivers preferring emotional support and reciprocity of care from their stroke survivors coded under the dyadic caregiver support type, caregivers have previously voiced their need to be appreciated by their care recipient as well [9, 36]. Moreover, caregivers who perceive limited mutuality of care in their relationship may be more likely to stop providing care [37].

Similar to the extended caregiver support system reported in our results, caregivers have been previously described as actively seeking partnerships with family members for getting assistance with household chores so that they can focus on their caregiving responsibilities. Though not explicitly describing the caregiving arrangements of the caregivers post-stroke, researchers have reported this social phenomenon of ‘seeking’, and defined it as ‘*carers’ efforts to better understand their role and to begin to establish some degree of ‘balance’ within their new and often confusing situation*’ [10].

Caregivers of stroke survivors have previously described the ‘*lack of personal resources*’ to address their caregiving related needs (e.g., information and educational needs) and fulfil the caregiving role post-stroke [38]. In some instances, they found it more productive to interact with peers to get information about the healthcare system and available services than approaching healthcare professionals [11]. Similarly, caregivers in our study reported the establishment of the empowering caregiver support system to address such informational, educational, and in some instances, financial needs by engaging peer caregivers who had been through a similar experience. Spouse caregivers have previously shared the importance of social relationships and support in an Australian context. They tend to prefer fellow caregiver support more as compared to support from family or close friends. They valued interactions with fellow caregivers as a means to share and learn new knowledge and skills to enable them to provide better care to their loved ones [39]. We can draw similarities with our finding that spouse caregivers prefer an empowering caregiver support system involving peer caregivers compared to a distributed caregiver support system involving sharing caregiving tasks with family and friends. A possible explanation may be that they do not want to burden their family members and may feel guilty engaging them in the care of their stroke survivors.

Along with describing the main caregiver support types, we also reported the related differences across different caregiver identities in our study. Authors have previously reported differences in the type of support needs of the caregivers. For instance, spouse caregivers relied on formal services like home care services to assist in physical caregiving tasks. In contrast, adult-child caregivers relied more on support groups to share their caregiving challenges [17]. We report slightly different findings, with spouse caregivers in our context expressing themselves as more central and autonomous in care provision, with limited support from different caregiving support systems. Moreover, in our study, adult-child caregivers did not express the need for support groups to share, possibly because they usually practised distributed caregiving, which may also provide an avenue for sharing among the co-caregivers. Another possible explanation for differences could be the care recipient population in both settings, i.e., people with dementia versus stroke survivors. Another difference was related to the use of formal services; specifically, participants in our study did not mention the use of such paid professional services. This could be related to greater prevalence and preference for informal caregiving support arrangements in our setting. Alternatively, there may be limited awareness of available formal services among stroke survivors and their caregivers. It has been reported that non-utilization of formal services by caregivers within Singapore is a common occurrence, potentially due to the lower perceived need for support, with FDWs as an alternative source of care arrangement.

Additionally, caregivers have been reported to have a negative perception of such services in terms of adequacy and quality [40]. Other reported reasons include lack of time, knowledge of available services and lack of perceived need [41]. From a cultural perspective, reliance on FDWs is more aligned with the filial responsibilities of family caregivers. It allows them to continue caring for their loved ones in the comfort of their homes compared to seeking formal services outside of the home environment. Additionally, past literature suggests the use of formal support services to be more common in caregivers in Western settings as compared to Asian settings [22]. A recent qualitative study including Chinese family caregivers of stroke survivors reported caregivers’ preference for restricted use of formal caregiving services based on higher self-reliance and a sense of self-sacrifice [23].

By describing the preferred caregiver support types across spouse and adult-child caregivers as extended/empowering/dyadic and distributed respectively, we have established the centrality of spouse caregivers in the post-stroke caregiving journey. They mainly seek emotional support from their loved ones and caregiving related information and skills from their peers, indicating the preferred auxiliary function of their support system. Along the same thread, a qualitative study in Scotland reported spouse caregivers’ preference to care for their stroke survivors rather than institutionalizing them [42]. This personal involvement in care provision of the spouse caregivers is similar to our finding of their surrounding support system being more peripheral in functionality, supporting spouse caregivers’ intimate involvement in care provision. Along the same lines, spouse caregivers in the past have been reported to be less willing to take help from others [35]. This may explain the relatively less intensive caregiver support system preference of spouse caregivers (i.e., extended or dyadic caregiving support system) compared to adult-child caregivers (i.e., distributed caregiving support system). By highlighting the differences in caregiver support system across different caregiver identities (i.e. spouse versus adult-child caregivers), we suggest considering caregiver identity as a surrogate for the surrounding support system and accompanying caregiving arrangements to provide context to the quantitative findings of caregiver identity being associated with outcomes of stroke survivors (e.g., rehospitalization post-stroke) [30, 43]. Future research efforts should focus on exploring the impact of different support systems on the caregiving experiences of caregivers from a sustainability perspective.

Following are the practical implications of our findings. Firstly, involving stroke survivor-caregiver dyads in training related to relationship-building skills to ensure the sustainability of the dyadic caregiver support system. The second practical implication is related to the adult-child caregivers’ preference of sharing caregiving tasks under the distributed caregiver support type compared to spouse caregivers. A possible explanation could be that the adult-child caregivers have other competing commitments, work or family related [19], which may necessitate developing a caregiving support system comprising of FDWs, with a more central caregiving role being assigned to them. Another possibility could be related to the relationship with the stroke survivor itself. Adult-child caregivers have previously reported a conflict in roles of being a child and caregiver simultaneously, being anxious and tense regarding negotiating privacy across the intimate care needs of their parents. Similar to this, it may be the case in our context, which explains heavy reliance on FDWs by adult-child caregivers compared to spouse caregivers [44]. The related practical implication would be for the healthcare providers practising family-centred care involving stroke survivors and their caregivers [45] to understand the type of caregiving support system surrounding the stroke survivor-caregiver dyad and engage them in collaborative healthcare delivery. For instance, under distributed type of caregiving support system, the healthcare provider should engage the main caregiver and ensure that the information and/or training is given to the right member of the support system (i.e., the one designated to perform the specific caregiving task) to prevent any breaks in care of the stroke survivor. The third practical implication is related to spouse caregivers’ preference for empowering caregiver support type involving their peers. Specifically, to provide more formalized avenues for the caregivers to interact with their peers and share experiences enabling them to care more optimally for their loved ones. Sharing sessions could be organized alongside outpatient clinic appointments so that caregivers can bank on the healthcare encounters of their stroke survivors to interact with peer caregivers. This would be especially useful for spouse caregivers who more frequently shared their preference for such interactions as compared to adult-child caregivers.

Our study has the following strengths. We are among the first, to the best of our knowledge, to provide a comprehensive account of different types of support systems surrounding the family caregivers of stroke survivors, providing insights into the preferences of spouse and adult-child caregivers. We had a diverse sample without any language related recruitment exclusions. Moreover, we captured the perspectives of both the stroke survivors and their caregivers in our study. We conformed to the trustworthiness criteria recommended for qualitative studies [29, 46], which adds to the dependability of our findings.

The following are some of the limitations of our study. Since we did not have access to patient health records, we could not capture information on stroke severity. Considering stroke severity may influence caregiving tasks, and in turn caregiving experience and support needs, we captured information on stroke recurrence as a proxy of stroke severity along with participant accounts of stroke symptoms and subsequent impairments. In spite of efforts to have representation of different types of caregivers, our sample was mainly limited to spouse and adult-child caregivers with limited representation of sibling (*N* = 2) or other (N = 2) caregivers. The profile of the caregiver sample is in line with the prevalence estimates of different caregivers reported in the stroke population in this setting (i.e., spouse and adult-child caregivers being the most common) [43]. Due to these recruitment related challenges, our findings would potentially be transferrable to spouse and adult-child caregiver populations only. Participant checking of research findings was not conducted since the participant identifiers (including contactable information) were not retained beyond data collection as per the study protocol.

## Conclusion

We have reported the different types of caregiver support systems of the caregivers of stroke survivors in an Asian setting, namely, dyadic, extended, distributed and empowering types. Additionally, we described the cultural influence on caregiving and the emergent findings of breaks in the care of stroke survivors and complex relationship dynamics. Our findings have illustrated caregiver identity (i.e., spouse or adult-child) as a surrogate marker for the differences in the caregiver support systems across both types of caregivers. While spouse caregivers preferred the dyadic and the extended support systems with more reliance on peers, adult-child caregivers preferred the distributed support system involving family members with paid helpers or FDWs playing a more central role. Spouse caregivers described themselves as more central and autonomous in post-stroke caregiving with a preference for a more peripheral role of the support system. Practical implications of our findings are provision of relationship-building skills to the stroke survivor-caregiver dyads to sustain dyadic caregiver support system, educating healthcare professionals practising family-centred care about tailoring engagement and management efforts to the caregiving arrangements of the caregivers, and creating formalized avenues for the caregivers to interact with their fellow peers and share experiences enabling them to care optimally for their loved ones.

## Supplementary Information

**Additional file 1.** COREQ Checklist.

## Data Availability

The data that support the findings of this study are available on request from the corresponding author. The data are not publicly available due to privacy or ethical restrictions.

## Abbreviation

FDWs: Foreign domestic workers
