# Supplementary material for: Support system diversity among family caregivers of stroke survivors: a qualitative study exploring Asian perspectives
Source: BMC Geriatr. 2021 Oct 25;21:594. doi: 10.1186/s12877-021-02557-4 (PMC8543837; doi:10.1186/s12877-021-02557-4)
Supplement: Supplementary file 1 — Additional file 1. COREQ Checklist. [file 12877_2021_2557_MOESM1_ESM.docx]

**Consolidated criteria for reporting qualitative studies (COREQ): 32-item checklist**

Developed from:

Tong A, Sainsbury P, Craig J. Consolidated criteria for reporting qualitative research (COREQ): a 32-item checklist for interviews and focus groups. *International Journal for Quality in Health Care*. 2007. Volume 19, Number 6: pp. 349 – 357

| **No. Item** | **Guide questions/description** | **Reported on Page #** |
| --- | --- | --- |
| **Domain 1: Research team and reﬂexivity** |  |  |
| *Personal Characteristics* |  |  |
| 1. Inter viewer/facilitator | Which author/s conducted the interview or focus group? | ST, AC, AF and ZL.  Page – 7-8 |
| 2. Credentials | What were the researcher’s credentials? E.g. PhD, MD | ST - PhD  SLC – MPH  AF - MPH  ZL – BSc  Page – 7-8 |
| 3. Occupation | What was their occupation at the time of the study? | ST – PhD candidate at SSHSPH, NUS  SLC – MPH student, SSHSPH, NUS  AF and ZL – Research Assistant, SSHSPH, NUS  Page - 8 |
| 4. Gender | Was the researcher male or female? | AT, SLC, AF and ZL: Female  Page -8 |
| 5. Experience and training | What experience or training did the researcher have? | ST is experienced qualitative researcher with prior experience across different qualitative projects.  SLC had basic training in qualitative research (MPH).  AF and ZL were new to qualitative research.  ST led qualitative methods and software training for rest. ST supervised early interviews.  Page – 7-8 |
| *Relationship with participants* |  |  |
| 6. Relationship established | Was a relationship established prior to study commencement? | No prior relationship between the interviewers and interviewees existed.  Page - 8 |
| 7. Participant knowledge of the interviewer | What did the participants know about the researcher? e.g. personal goals, reasons for doing the research | Potential participants were informed about the study and its purpose and those agreeable were enrolled for interviewing.  They were further informed about the study by interviewers with sharing of study information sheet (approved by the Institutional Ethics Review Board).  Page - 6 |
| 8. Interviewer characteristics | What characteristics were reported about the inter viewer/facilitator? e.g. Bias, assumptions, reasons and interests in the research topic | Participants knew about the work scope of the interviewers.  Page - 6 |
| **Domain 2: study design** |  |  |
| *Theoretical framework* |  |  |
| 9. Methodological orientation and Theory | What methodological orientation was stated to underpin the study? e.g. grounded theory, discourse analysis, ethnography, phenomenology, content analysis | The study was based on the principles of qualitative description, in which qualitative approach is adopted to get participants’ experiences and perceptions of the phenomenon being studied.  Further researchers used thematic analysis to analyze the data.  Please refer Page – 8-9 for further details on data analysis. |
| *Participant selection* |  |  |
| 10. Sampling | How were participants selected? e.g. purposive, convenience, consecutive, snowball | Purposive sampling was used.  Page - 6 |
| 11. Method of approach | How were participants approached? e.g. face-to-face, telephone, mail, email | Participants were approached face-to-face in outpatient rehabilitation setting and outpatient clinic setting, while email was sent for the support organization for stroke survivors and their caregivers.  Page - 6 |
| 12. Sample size | How many participants were in the study? | 61 total participants: 35 caregivers and 26 stroke survivors.  Page - 10 |
| 13. Non-participation | How many people refused to participate or dropped out? Reasons? | Not captured.  The non-participation rate was not captured since the preliminary screening and assessment for eligibility criteria was done by staff at the outpatient clinic and outpatient rehabilitation setting who had limited availability of time and resources. Additionally, for the support organization we were only contacted by participants who were willing to participate.  Page - 10 |
| *Setting* |  |  |
| 14. Setting of data collection | Where was the data collected? e.g. home, clinic, workplace | Participants were either interviewed at their homes, or outpatient clinic setting or in a community setting of participant preference.  Page - 7 |
| 15. Presence of non-participants | Was anyone else present besides the participants and researchers? | Non-participants were not present when interviews were conducted.  Page - 7 |
| 16. Description of sample | What are the important characteristics of the sample? e.g. demographic data, date | Information about the demographic information was collected for all participants and is presented in **Table 3** |
| *Data collection* |  |  |
| 17. Interview guide | Were questions, prompts, guides provided by the authors? Was it pilot tested? | Interview guide was used to collect data and it was pilot tested.  Topic guide is presented in **Table 1**. |
| 18. Repeat interviews | Were repeat inter views carried out? If yes, how many? | There were no repeat interviews.  Page – 7 |
| 19. Audio/visual recording | Did the research use audio or visual recording to collect the data? | Interviews were audio-recorded and transcribed verbatim.  Page - 8 |
| 20. Field notes | Were ﬁeld notes made during and/or after the inter view or focus group? | Field notes were taken, and memos were written post interviews.  Page - 8 |
| 21. Duration | What was the duration of the inter views or focus group? | Interviews generally lasted from 28 to 58 minutes.  Page - 7 |
| 22. Data saturation | Was data saturation discussed? | Yes, data collection was stopped once the researchers observed recurring themes emerging.  Page -7 |
| 23. Transcripts returned | Were transcripts returned to participants for comment and/or correction? | Transcripts were not returned to participants since participant identifiers and contactable information was not retained beyond the interview stage. Page - 8 |
| **Domain 3: analysis and ﬁndings** |  |  |
| *Data analysis* |  |  |
| 24. Number of data coders | How many data coders coded the data? | ST was the primary coder, with a subset of interviews being coded by AC. Coding consistency was checked by coders via discussions among the team.  Page -8 |
| 25. Description of the coding tree | Did authors provide a description of the coding tree? | The authors provided a description of main themes and sub-themes along with providing a diagrammatic summary based on synthesis of themes.  Page - 10 |
| 26. Derivation of themes | Were themes identiﬁed in advance or derived from the data? | Themes were related to subject matter in the topic guide, along with emerging themes which were inductively derived from data.  Page – 10-11 |
| 27. Software | What software, if applicable, was used to manage the data? | NVivo 12 qualitative data analysis software.  Page - 9 |
| 28. Participant checking | Did participants provide feedback on the ﬁndings? | Participant checking of research findings was not conducted since the participant identifiers (including contactable information) were not retained beyond data collection as per the study protocol.  Page - 28 |
| *Reporting* |  |  |
| 29. Quotations presented | Were participant quotations presented to illustrate the themes/ﬁndings? Was each quotation identiﬁed? e.g. participant number | Yes, quotations were identified by participant number and whether the participant was a patient or caregiver.  Please refer to the results section of the manuscript |
| 30. Data and ﬁndings consistent | Was there consistency between the data presented and the ﬁndings? | We have attempted to present our findings in a clear manner, consistent with the data collected.  Please refer to the results section of the manuscript |
| 31. Clarity of major themes | Were major themes clearly presented in the ﬁndings? | Yes  Please refer to the results section of the manuscript |
| 32. Clarity of minor themes | Is there a description of diverse cases or discussion of minor themes? | Yes, the authors have presented description of all major and minor themes within the manuscript  Please refer to the results section of the manuscript |
| Abbreviations: ST = Shilpa Tyagi, SLC= Audrey Swee Ling Chan, AF= Aysha Farwin, ZL=Zunairah Binti Lukman, SSHSPH, NUS = Saw Swee Hock School of Public Health, National University of Singapore. | | |
